# Group Membership Modulates the Neural Circuitry Underlying Third Party Punishment

**DOI:** 10.1371/journal.pone.0166357

**Published:** 2016-11-11

**Authors:** Rosalba Morese, Daniela Rabellino, Fabio Sambataro, Felice Perussia, Maria Consuelo Valentini, Bruno G. Bara, Francesca M. Bosco

**Affiliations:** 1 Department of Psychology, Center for Cognitive Science, University of Turin, Turin, Italy; 2 Faculty of Communication Sciences, Università della Svizzera Italiana, Lugano, Switzerland; 3 Department of Psychiatry, Schulich School of Medicine and Dentistry, University of Western Ontario, London, Ontario, Canada; 4 Department of Experimental and Clinical Medical Sciences (DISM), University of Udine, Udine, Italy; 5 Department of Neuroradiology, Hospital-Città della Salute e della Scienza di Torino, Turin, Italy; 6 Neuroscience Institute of Turin, University of Turin, Turin, Italy; University of the Basque Country, SPAIN

## Abstract

This research aims to explore the neural correlates involved in altruistic punishment, parochial altruism and anti-social punishment, using the Third-Party Punishment (TPP) game. In particular, this study considered these punishment behaviors in in-group vs. out-group game settings, to compare how people behave with members of their own national group and with members of another national group. The results showed that participants act altruistically to protect in-group members. This study indicates that norm violation in in-group (but not in out-group) settings results in increased activity in the medial prefrontal cortex and temporo-parietal junction, brain regions involved in the mentalizing network, as the third-party attempts to understand or justify in-group members’ behavior. Finally, exploratory analysis during anti-social punishment behavior showed brain activation recruitment of the ventromedial prefrontal cortex, an area associated with altered regulation of emotions.

## Introduction

The ability to cooperate is one of the features that characterizes human beings and represents a crucial step in social evolution: it shapes the norms that allow the creation of groups and the development of institutions [1–2]. In social groups, adherence to shared rules and social norms is essential for survival, and transgressors are punished for cheating even in the absence of any personal benefit [3]. Fehr and Gächter [3] defined this form of behavior as altruistic punishment, (see also [4–5]). Various economic games have investigated altruistic punishment, revealing its crucial role in maintaining cooperation among participants [6–11]. A well-studied case is the Third-Party Punishment (TPP) game. In the TPP game a third party (player C) observes an economic interaction between player A and player B. Player A can share part of his own amount of money with Player B, who has a passive role and can only accept player A's proposal. Player C can spend part of his own amount of money to punish player A's (unfair) behavior, even though he is not directly affected by Player’s A moves.

There is a wealth of neuroimaging literature that describes the neural networks underlying TPP. Increased activation of the dorsolateral prefrontal cortex has been observed among participants making decisions about punishing defectors of social norms [12–13], while increased activation of the thalamus, nucleus accumbens, anterior cingulate cortex, insula [11], and caudate nucleus have been found to reflect the satisfaction of a third-party when punishing defectors [14–15]. The latter set of brain regions has been identified as the reward system [11]. Furthermore, some researchers have also associated punishing behavior during economic games with increased activation of the mentalizing network (e.g. medial prefrontal cortex and temporo-parietal junction), salience network (e.g. amygdala, insula) and central-executive network (e.g. dorsolateral prefrontal cortex, posterior parietal cortex). In particular, the dorsolateral prefrontal cortex seems to be important in the implementation of intention-based economic cooperation, and also has an important role in moral judgment, understanding and determining moral responsibility, and assigning appropriate punishment [16].Experimental evidence gained during cooperative vs. competitive processes has pointed to differences in interactions with in-group members, i.e. participants belonging to the same group, and out-group members, i.e. participants belonging to different groups [17–18]. In particular, people tend to cooperate more in in-group than in out-group settings [19–20]. In social interactions between different groups people tend to protect or favor, even without any personal gain, their own group members at the expense of those of other groups [21–22]. Bernhard et al. defined this in-group and out-group difference as parochial altruism [23] (see also [24–25]. In a previous study Rabellino et al. [26] examined this behavior in in-group and out-group game settings where the groups differ for nationality (Chinese or Italian). Behavioral results indicated that altruistic punishment behavior emerges as the tendency to protect in-group victims of unfair behavior (see also [23]). The first study that explored the neural correlates of parochial altruism [27], in which in-group and out-group conditions were composed by officer candidates randomly assigned to different platoons (groups) at the beginning of the training course, revealed that the punishment of members belonging to the same group was associated with increased activity and connectivity within the mentalizing system. Notably, this system is involved in predicting other people’s behavior and inferring their mental states, such as thoughts, beliefs, desires, and intentions [28–32].

Goette et al. explored the existence of an opposite and apparently paradoxical behavior, namely anti-social punishment, which is the tendency to spend one's own money even to punish cooperative behavior [33]. In order to explain such unusual and puzzling behavior Herrmann et al. suggested that people who have acted unfairly in the past and have been punished for that reason, may use antisocial punishment as a form of revenge on cooperators [34]. Alternative explanations may entail specific personality traits: individuals with a competitive personality would tend to increase the drive to maximize other individuals’ payoffs. However, the interpretation of antisocial punishment is far from conclusive as the motivations behind this paradoxical behavior have yet to be understood. Owing to the difficulty of reconciling this behavior with the classical models of evolution of cooperation, it was not included in earlier models of social behavior [34–35].

Finally, racial prejudice can also influence economic decision-making in the context of in-group and out-group settings. Behavioral studies [36–37] have shown that social group cues, such as a partner's gender or ethnicity, can shape trust decisions in economic games. In addition, empathy can contribute to economic decision-making [38], and can facilitate prosocial behavior in economic games in the form of greater cooperation or generosity [39–41].

In [26] the authors investigated and analyzed only the behavioral aspects of altruistic punishment, parochial altruism and antisocial punishment in an in-group and out-group game setting. The aim of the present study was to focus our attention on the neural substrates underlying altruistic punishment, parochial altruism, and anti-social behavior in in-group and out-group settings. The novelty and interesting aspect of this study is that the group settings were created using the participants’ real nationality group membership:, i.e. Italian vs. Chinese. Baumgartner et al. [27] only investigated the neural circuitry of the impact of group membership, i.e. parochial altruism, on social norm enforcement, in which in-group and out-group conditions were composed by officer candidates randomly assigned to different platoons (groups) at the beginning of the training course. The present paper is the first to also investigate the neural activity of brain areas underlying altruistic and anti-social punishment, in addition to parochial altruism. Furthermore, differently from Baumgartner et al.[27], in our study group membership was based on the participant’s nationality.

At behavioral level we expected to replicate the results obtained in a previous study [26]. In particular, we expected that: i. player C would punish unfair behavior by the dictator (player A), i.e., altruistic punishment; ii. player C would invest more money to punish the dictator’s unfair behavior if player B was an in-group member rather than an out-group member, i.e., parochial altruism; iii. player C would, albeit to a lesser extent, spend resources to punish the cooperator, i.e., anti-social punishment.

In addition, in the present study we investigated the neural correlates involved in the above-mentioned punishment behaviors. Following on previous neuroimaging studies of behavior during TPP economic games [11–15], [42] we expected that: iv. altruistic punishment would be associated with activation of the reward system; v. parochial altruism would recruit brain regions involved in the mentalizing system. Lastly, for exploratory purposes, in the case of participants who adopted this kind of behavior, as occurred in [26], we also investigated the neural correlates underlying antisocial behavior in both the in-group and out-group settings.

Finally, given that previous studies have shown that empathy [38] and racial prejudice [36–37] have a role in decision-making processes, we explored the role of these factors in explaining the behaviors in question.

## Methods

### Participants

Twenty-three Italian male volunteers (age 24.56 ± 1.87 years) were recruited from among undergraduate students at the University of Turin. We chose to include males only, in order to avoid the effect of gender differences on cooperative choices, see [43–44]. We also excluded students of Psychology or Economics since they might already have been familiar with economic games. Two participants were excluded from the study because of excessive movement (>2 mm) during the fMRI scans. The experimental subjects were right-handed according to the Edinburgh Handedness Inventory [45] and did not have a history of neurological or psychiatric disorders. None of them was taking medication affecting the central nervous system or had any previous experience of economic games. After a detailed presentation of the study all participants gave their written informed consent. The study was approved by the Bio-Ethics Committee of the University of Turin.

### Procedure and measures

We used the TPP paradigm. In particular, we added a third player (C) to a classic Dictator Game, such as in Strobel et al. [11]. In a Dictator Game two players interact: player A, the dictator, and player B, the receiver. In our experiment player C watched a video of a modified Dictator Game acted out by player A and player B in the MR scanner waiting room. Here, the experimenter read English instructions to the players, two Italian and two Chinese (confederates of the experimenters) on how to play the game. We controlled that the experimental subjects were not aware that the other participants were confederates of the experimenters. We tested this aspect with post-experiment open questions in which we asked the experimental subjects to give their opinions about the other players (for example “What do you think about the other players?” “Do you think the other players know each other?”). None of the experimental subjects mentioned the possibility of the Chinese participants being confederates of the experimenter.

After the debriefing, player C read the instructions on his role in the TPP game and answered a questionnaire to assess his understanding of the game. TPP training was performed outside of the scanner to ensure that participants understood the game. A short practice session in the scanner was administered to familiarize the participants with the response-recording system. The trial games were pseudo-randomized (software E-Prime 2.0, 2007, Psychology Software Tools).

**Fig 1:**
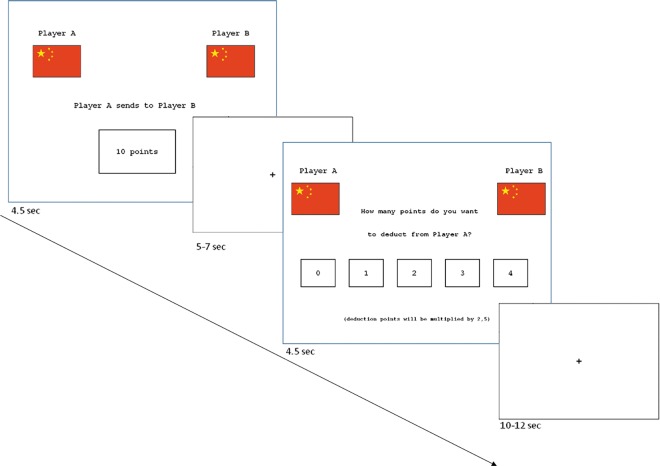
An example of a trial used in the present study. In this case Players A and B belonged to the *out-out group condition* (OUT- OUT).

Two functional runs of the TPP were administered to each subject, with an anatomical scan in between. In each run, subjects took part in 48 trials (repetitions of the game) in an event-related design, composed of 24 fair and 24 unfair conditions. The order of runs was counterbalanced across subjects. Each trial included: player A's move (fair or unfair) presented for 4.5 sec., jittered fixation cross lasting 5 or 7 sec. and player C’s decision. Subjects had 4.5 seconds to decide on whether to punish player A using a keypad with a scale from 0 to 4 MU (see Fig 1 for an example). Each subject’s decision was displayed on the screen. Trials were separated by jittered inter-trial intervals (fixation cross, duration = 10 or 12 sec.). The participants did not know the identity of players A and B, but only their nationality, which was represented by the Italian or Chinese flag, used to manipulate in-group and out-group settings and to identify participants’ membership. National flags may represent a contextual situation where a non-social stimulus can effectively become equivalent to a social one [46].

Four different player combinations were displayed depending on their in-group and out-group membership relative to player C: the in-in group condition (IN-IN), where players A and B belonged to the same group as player C; the out-group condition (OUT-OUT), where player A and player B belonged to the out-group; (IN-OUT), player A belonged to the in-group and player B belonged to the out-group; (OUT-IN), player A belonged to the out-group and player B to the in-group.

At the beginning of each trial player A had an initial endowment of 20 Monetary Units (MU) that he could decide to give (or not) to player B. Also player C at the beginning of each trial had an initial endowment of 4 MU. In particular the experimenter told the participants that “*Player A could send to Player B some*, *all*, *or none of his sum”* and that “*Player C was free to assign a number of deduction points from his endowment*, *from zero to 4*, *to Player A”*. We highlight that the experimenter, in order to avoid any bias, never defined Player A’s behavior as “fair” or “unfair”. Following Strobel et al. [11] and Fehr & Fischbacher [7], the trials were classified as unfair (≤ 7 MU) or fair (≥8 MU) based on the amount of MU given by player A to player B. The fair/unfair cut-off was not revealed to subjects prior to the experiment. For every MU spent by player C, 2.5 MU were subtracted from Player A’s payoff: for example, if player C spent all of his 4 MU (the maximum punishment) to sanction player A’s behavior, 10 MU would be subtracted from player A’s payoff. In the experiment, player A and player B belonged to the Italian or Chinese group. After the fMRI session, the participants were administered a version of the Implicit Association Test (IAT) [47–48] to evaluate implicit race bias towards European and Asian faces. During testing, participants' reaction times for two critical sets of trials were recorded. In the first set, faces of people in the participant’s own racial group were paired with pleasant words (congruent trials), and in the second set with unpleasant words (in-congruent trials.) The difference in reaction times between congruent and in-congruent trials measures implicit race bias (IAT D-score). Participants’ empathy skills were measured using the Interpersonal Reactivity Index (IRI) [49].

Participants received a lump sum payment of 20 euros for taking part in the experiment, plus the money earned during the TPP game (ranging from 0 to 20 euros). The average earnings were 32.20 euro per participant.

#### Data acquisition and analysis

Blood-oxygen-level-dependent (BOLD) responses were measured while participants performed the TPP using a Signa 1.5 T head scanner (GE Healthcare, Milwaukee, Wisconsin) at the CTO Hospital in Turin. Head motion was restricted using soft padding around the head. Functional data were acquired using T2*-weighted Echo-Planar Images (EPI) (TR = 2.25s, TE = 50ms, slice-matrix = 80×80, slice gap = 0.28 mm, field of view (FOV) = 21 cm, flip angle = 90°, slices aligned on the AC-PC line) during two functional runs. The first four volumes of each run were discarded to allow for equilibration of T1 saturation effects. In between the fMRI runs T1-weighted anatomical images (Sag 3D BRAVO) were also acquired (with resolution 1 mm3; TR = 7.92 ms; TE = 2.4 ms; TI = 910 ms; BW = 195 Hz/Px; α = 15°).

#### Behavioral data

Behavioral data analyses were carried out using SPSS 20.0 (SPSS Inc., Chicago, IL, USA) with α set to p<0.05 (two tailed). Altruistic punishment was studied by measuring the nationality factor at four levels with respect to player C’s nationality (in-group condition (IN-IN), players A and B belonged to player C’s group; the out-group condition: (OUT-OUT), player A and player B belonged to the out-group; (IN-OUT), player A belonged to the in-group and player B belonged to the out-group; (OUT-IN), player A belonged to the out-group and player B to the in-group). We also calculated a repeated-measures ANOVA with fairness at two levels (*fair; unfair*) and Group Membership at four levels (IN-IN), (OUT-OUT), (IN-OUT) and (OUT-IN). To test parochial altruism behavior we used a repeated-measures ANOVA with the nationality factor at four levels: (IN-IN), (OUT-OUT), (IN-OUT) and (OUT-IN) during the unfair condition. We then conducted a post hoc paired t-test. To explore antisocial punishment behavior we applied a repeated-measures ANOVA with the nationality factor at four (IN-IN), (OUT-OUT), (IN-OUT) and (OUT-IN) during the *fair* condition. In case of significant multivariate effects, post hoc paired t-tests were conducted. In order to investigate the relationship between individual characteristics, such as empathy and implicit race bias, and punishment behavior we used the Pearson Correlation.

#### FMRI data

Data were analyzed using SPM8 (Wellcome Department of Cognitive Neurology, London, UK) implemented in Matlab (Mathworks, Cherborn, MA, USA). All functional images were spatially realigned to the first volume and coregistered to the mean image. All images were normalized to the MNI (Montreal Neurological Institute) space and smoothed with an 8 mm Gaussian Kernel, with an additional 6 mm smoothing at the first level for parochial altruism (considered the complexity of in-group vs. out-group contrasts during this condition).

After preprocessing we applied General Linear Modelling (GLM) [50] for statistical analysis. At the first level, each trial was modeled by convolving a stick function with a hemodynamic response function. The GLM consisted of a set of 17 regressors: four categorical regressors for social group membership: (IN-IN), (IN-OUT), (OUT-IN) and (OUT-OUT) conditions were included for each fairness condition (fair and unfair); two categorical regressors for player C’s decisions (punishment and no punishment), six parametric regressors for motion extent and one regressor for the nationality check trial.

At the second level, to investigate the neural correlates of altruistic punishment behavior, we performed a one-sample t-test of the contrast unfair punishment *vs*. unfair no punishment across all the participants. To test our hypotheses on the network areas recruited during parochial altruism we used a full factorial model with four levels during the unfair condition for the four group settings, (IN-IN), (IN-OUT), (OUT-IN), (OUT-OUT) across all the participants. To explore neural correlates of anti-social punishment, we performed a separate one-sample t-test of the contrast *fair punishment vs*. *fair no punishment*. This analysis was limited to those participants (n = 10) who showed this particular and puzzling behavior. For altruistic punishment and parochial altruism we used a small volume correction (SVC) with a sphere of 10 mm radius centered on coordinates based on previous neuroimaging studies on punishment. Specifically,for altruistic punishment, we focused on: the ventromedial prefrontal cortex (10 mm radius sphere centered on x = 2 y = 54 z = – 4 [14], the ventral tegmental area (x = 8 y = -18 z = -10 [51], the anterior cingulate cortex (x = 0 y = 44 z = 10 [51], the left (x = 40 y = 16 z = -2 [11]) and right anterior insula (x = 32 y = 24 z = -4 [11]), for dorsolateral prefrontal cortex we applied results reported by Knoch et al. [12] x = 39 y = 37 z = 22, Talairach coordinates converted to MNI x = 43 y = 41 z = 25 using http://www.sdmproject.com/). For Parochial Altruism we focused on: the left (x = -3 y = 54 z = 28 [52]) and right medial prefrontal cortex (x = 6 y = 50 z = 31 [52]), the right (x = 47 y = −61 z = 39 [52]) and left temporal-parietal junction (x = -46 y = -63 z = 41 [52]), and the caudate nucleus (5 mm radius sphere centered on x = -14 y = 12 z = 8 based on [53] and [54]. All results are presented within a statistical threshold of p<0.05 family-wise error corrected for multiple comparisons. For the exploratory whole brain analyses of anti-social punishment only, we used a liberal threshold of p< 0.005 uncorrected and a cluster size of k≥10.

## Results

### Behavioral results

In the altruistic punishment analysis there was a main effect of fairness (F_(1,20)_ = 35.697, p < .001) with participants investing more MU to punish the *unfair* condition as compared to the *fair* condition (post hoc paired t-test: p < .001). There were no significant effects of players’ group membership (F_(1,20)_ = .002, p = .969) and no group by membership interaction (F_(1,20)_ = 1.459, p = .244).

In the *parochial altruism analysis* there was a main effect of nationality (F _(3,60)_ = 3.194, p < .049). Follow-up t-tests indicated that the participants spent more MU on punishment in unfair (IN-IN) than in unfair (IN-OUT) conditions (t_(21)_ = 1.994, p = .01). No significant differences emerged from other comparisons.

Finally, the anti-social punishment analysis revealed a significant effect of nationality (F_(3,60)_ = 3.804, p < .015). Post-hoc paired t-tests indicated that in the *fair* condition participants tended to punish more frequently in (OUT-OUT) settings than in (OUT-IN) ones (t_(21)_ = 1.980, p = .008).

### FMRI results

#### Altruistic Punishment

**Fig 2:**
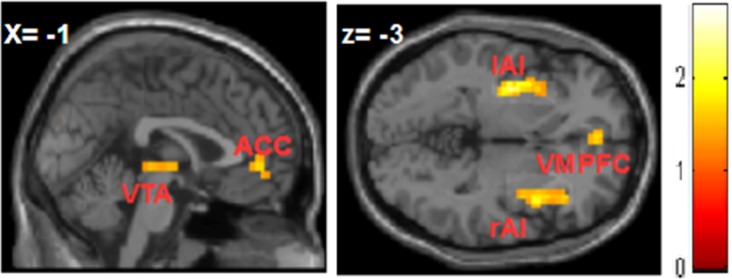
FMRI results: Altruistic Punishment. Brain activation maps during altruistic punishment behavior for the *punishment vs*. *no punishment* contrast: (A) ventral tegmental area (VTA), anterior cingulate cortex (ACC);(B) right and left insula (rAI; lAI), ventromedial prefrontal cortex (VMPFC).

In the unfair condition we found significant increased activation during punishment vs. no punishment in the following brain areas: ventral tegmental area (VTA), right and left anterior insula (rAI; lAI), anterior cingulate cortex (ACC), ventromedial prefrontal cortex (VMPFC) (see Fig 2 and Table 1).

**Table 1:** Brain regions revealed by contrasts of interest.

Region of activation	Coordinates			Z-score	p-value
	X	Y	Z		
**Altruistic punishment** *punishment > no punishment*					
ventromedial prefrontal cortex	-0.7	48	-2	3.62	0.005
ventral tegmental area	2	-20	-2	3.33	0.006
anterior cingulate cortex	2	45	4	3.54	0.011
L anterior insula	-33	9	-2	3.66	0.008
R anterior insula	38	15	-2	3.79	0.005
**Parochial Altruism** *unfair in-group condition (IN-IN) > unfair out-group condition (IN-OUT)*					
L medial prefrontal cortex	-10	58	34	2.99	0.019
R medial prefrontal cortex	12	55	28	3.17	0.012
R temporal-parietal junction	48	-56	40	6.77	0.000
L temporal-parietal junction	-40	-59	34	6.06	0.000
caudate nucleus	-10	15	10	2.29	0.040
**Antisocial punishment** *punishment > no punishment*					
Ventromedial prefrontal cortex*	2.5	45	4	3.91	0.000

Peak activity coordinates are given in MNI space.

Altruism punishment and parochial altruism were analyzed using a small volume correction (SVC) with a sphere of 10 mm radius (5 mm radius for the caudate nucleus) centered on the reported coordinates with a statistical threshold of p<0.05 family-wise error corrected for multiple comparisons.

* Liberal threshold of p< 0.005 uncorrected and a cluster size of k≥10.

#### Parochial altruism

**Fig 3:**
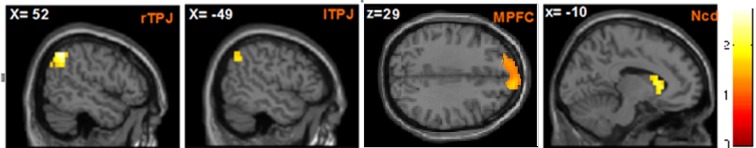
FMRI results: Parochial altruism. When player C observes unfair behavior in the *in-in group condition* (IN-IN) the recruitment of the mentalizing system, the medial prefrontal cortex (MPFC), and the right and left temporal-parietal junction (rTPJ and lTPJ) and, in addition, of the caudate nucleus (CN) were observed.

Neural activity increased bilaterally in the medial prefrontal cortex and temporo-parietal junction and the caudate nucleus (CN, see Fig 3 and Table 1) in the IN-IN condition as compared to IN-OUT.

#### Antisocial punishment

**Fig 4:**
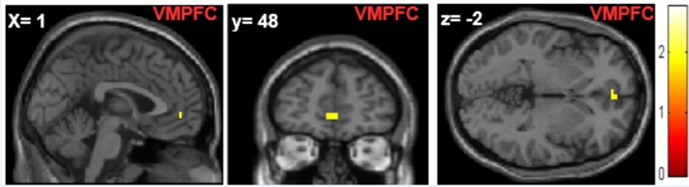
FMRI results: Antisocial punishment. Brain activation recruitment of the ventromedial prefrontal cortex during antisocial punishment behavior for the *punishment vs*. *no punishment* contrast in the in-group and out-group settings.

Results showed increased brain activity in the ventromedial prefrontal cortex (VMPFC) in the fair condition for the contrast punishment vs. no punishment (Fig 4 and Table 1).

### Psychological traits

Lastly, to evaluate implicit race bias towards European and Asian faces we analyzed the differences of reaction times between congruent and in-congruent trials measuring implicit race bias (IAT-D score). As mentioned previously, during testing, participants' reaction times for two critical sets of trials were recorded. In the first set, faces of people in the participant’s own racial group were paired with pleasant words (congruent trials), and in the second set with unpleasant words (in-congruent trials.) The difference in reaction times between congruent and in-congruent trials measures implicit race bias (IAT D-score). Scores of this index range from -2 to +2. A positive IAT-D score indicates that participants associated pleasant words and own racial group faster than unpleasant words, so implicit preference for own racial group. In the present study participants showed a slight to moderate preference towards their own group (IAT-D score = .34;(a value of ±0.15 counts as a break point for a "slight", ±0.35 for a "moderate", and ±0.65 for a "strong" association; see references [47–48]). None of the correlations performed between the IAT and each experimental condition yielded significant results (all rs ≥ .194, all ps ≤ .398), for detailed results see S1 Table in Supporting Information. The number of punishment events for unfair behavior showed a positive correlation with scores for empathy (IRI questionnaire, r = .465; p = .034) for both the in-group and out-group settings.

## Discussion

In the present study we investigated the behavioral and neural correlates of third party punishment. In particular, we focused on how in-group vs. out-group nationality membership modulates the neural circuits underlying decision-making during the TPP economic game.

At behavioral level, our results confirm the altruistic punishment behavior, in line with the relevant literature [3], [7], [26], [55], in both the in-group and out-group settings. Consistently with previous fMRI studies [11],[14–15] which indicated the recruitment of reward system areas during altruistic punishment behavior, our fMRI results reveal increased activation in the VTA, rAI, lAI, ACC and VMPFC; in particular the mibrain dopamine system and the VTA play a pivotal role in motivation and reward. Cohen et al. [56] and Strobel et al. [11] suggested that activation of the insula during punishment could be associated with processing of disgust [57–58]. Craig proposed a model of insula functioning, which entails its integration with the ACC into a complementary system, involved in voluntary motivation, or agency, and interoception of bodily conditions [59]. Previous studies have suggested that increased activity in the insula and the ACC reflects the interoceptive awareness of disgust evoked by the violation of social or moral norms [60]. Furthermore, previous studies have indicated the involvement of the dorsolateral prefrontal cortex in the implementation of cognitive control and its regulatory role in altruistic punishment [61], [11]. However, we did not observe the recruitment of the dorsolateral prefrontal cortex. A possible explanation for this finding could be that the experimental task used in the present study, aimed at attaining minimal cognitive components, resulted in a reduced engagement of the brain regions involved in attention processing, compared to those mentioned above. Another possible explanation for this negative finding could lie in the fact that in the in-group and out-group settings participants might make their choices without the recruitment of the dorsolateral prefrontal cortex for implementing intention-based economic cooperation, or moral judgment, understanding and determining moral responsibility, and assigning appropriate punishment.

In this study, behavioral and neural experimental evidence suggest that the satisfaction gained by punishing norm violations is the underlying motive for altruistic punishment behavior. In particular, our results show that satisfaction following the punishment of unfair behavior is felt in both in-group and out-group settings.

As for parochial altruism, we found that, in line with in-group favoritism [62], [20], participants were prone to protect their own group members by delivering harsh punishment to out-group members [26], [27]. This behavior resulted in increased activity within brain regions involved in mentalizing processes, including the MPFC and bilateral TPJ, and this change was greater when comparing the in-group condition (IN-IN) with the out-group (OUT-IN) condition. This result seems to suggest that when player C watches player A acting unfairly, he tries to understand the intentions or goals behind the in-group member’s unfair behavior in order to justify it. Our results are in line with the relevant literature showing that these two brain regions are key components of the mentalizing network, involved in inferring goals, intentions, desires, as well as more enduring dispositions of others [29–32], [27], [63–65], [28]. In addition to this network, we observed increased activity of the caudate nucleus. This brain structure has been associated with reward processing in several neuroimaging studies [66–68].

Finally, antisocial punishment is another important aspect emerging during TPP that can be modulated by group membership. In line with the findings of Bortolotti et al. [69], who reported considerable antisocial punishment behavior among Italian subjects, our behavioral results showed that player C punished fair behavior in the out-group condition more often when players A and B, both belonged to the out-group, as compared to when player A belonged to the out-group and player B to the in-group. These results might suggest a form of protection across in-group members, another possible explanation is that our task and experimental procedure may have evoked this competitive behavior in-group setting. This behavioral result was associated with increased activity of the ventromedial prefrontal cortex, which is a brain region pivotal for social decision-making [70–72]. Patients with lesions in the VMPFC have been found to show behavioral impairments affecting decision-making abilities and emotion processing during social tasks, including moral judgment and economic games: in particular, such patients make more irrational economic decisions compared to controls [73], [70], [72]. Moretti et al. showed that the VMPFC represents the subjective value or desirability of future outcomes during social decision-making in economic tasks [74]. Notably, lesions in the VMPFC have been associated with impairments in making value-based decisions in general [73]. Other researchers have suggested that VMPFC deficits are associated with altered regulation of emotions [70]. The activation of the VMPFC emerged from this exploratory analysis may follow on from both the regulation of emotions and making value-based decisions during punishment of cooperative behavior; however future studies are needed to ascertain the contribution of each of these processes.

As far as the personal characteristics of the individuals participating in the present study are concerned, we found that participants who tended to punish unfair behavior more often, irrespective of group membership, showed a positive correlation with scores of empathy (IRI questionnaire). Previous literature has suggested that empathy is an important factor in decision-making processes [39]. Several studies have indicated that empathy facilitates prosocial behavior towards others in the setting of economic games, in the form of greater cooperation or generosity [39–41]. Indeed, when people have a greater motivation towards establishing relationships with others this make them more likely to choose economic decisions benefiting others [75]. These findings suggest that empathy play an important role in cooperative behavior and may promote altruistic punishment behavior.

Finally, the participants showed a slight to moderate preference for their own racial group; therefore, since in the present investigation racial and nationality belonging collapse, this variable could have influenced our results about parochial altruism and antisocial punishment in relation to preference for one’s own nationality group.

A limitation of the present study is the small size and gender of the sample. In addition we cannot exclude that the use of flags could have affected participants’ behavior. In future, it would be interesting to use a larger sample also including female participants and including real face to face interactions to enable the generalization, or otherwise, of our results and further investigate the puzzling anti-social punishment behavior we observed.

In conclusion, in line with the relevant literature this study confirms the role of punishment behavior and the recruitment of reward systems during altruistic punishment behavior in both in-group and out-group settings. Moreover, our findings suggest a role of the mentalizing system and caudate nucleus during the observation of unfair behavior in the in-group setting.

These results are in line with the evolution theory according to which cultural aspects (knowledge, norms, language, beliefs, etc.) play a key role in maintaining in-group cooperation [76]. However, the relationships between in-group and out-group membership and their coevolution in punishment behavior are still unclear, therefore these aspects would need further examination.

## Supporting Information

S1 Table Correlations between IAT score and punishment behavior. (DOC)
