# Supplementary material for: Group Membership Modulates the Neural Circuitry Underlying Third Party Punishment
Source: PLoS One. 2016 Nov 11;11(11):e0166357. doi: 10.1371/journal.pone.0166357 (PMC5106004; doi:10.1371/journal.pone.0166357)
Supplement: S1 Table — (DOC) [file pone.0166357.s001.doc]

**S1 Table. Correlations between IAT score and punishment behavior**

|  | | *Altruistic Punishment* | | *Antisocial Punishment* | | | | *Parochial Altruism* | | | |
| --- | --- | --- | --- | --- | --- | --- | --- | --- | --- | --- | --- |
|  | | FAIR | UNFAIR | FAIR IN-IN | FAIR IN-OUT | FAIR OUT-IN | FAIR OUT-OUT | UNFAIR IN-IN | UNFAIR IN-OUT | UNFAIR OUT-IN | UNFAIROUT-OUT |
| IAT | Pearson Correlation | ,008 | ,133 | ,068 | ,023 | -,044 | -,003 | ,046 | -,002 | ,059 | ,194 |
|  | Sign. (2-tailed) | ,971 | ,567 | ,770 | ,921 | ,850 | ,989 | ,843 | ,994 | ,801 | ,398 |
|  | N | 21 | 21 | 21 | 21 | 21 | 21 | 21 | 21 | 21 | 21 |

|  |
| --- |
|  |
